# Multi-energy spectral photon-counting computed tomography (MARS) for detection of arthroplasty implant failure

**DOI:** 10.1038/s41598-020-80463-2

**Published:** 2021-01-15

**Authors:** Lawrence Chun Man Lau, Wayne Yuk Wai Lee, Anthony P. H. Butler, Alex I. Chernoglazov, Kwong Yin Chung, Kevin Ki Wai Ho, James Griffith, Philip H. Butler, Patrick Shu Hang Yung

**Affiliations:** 1 Department of Orthopaedics and Traumatology, Faculty of Medicine, The Prince of Wales Hospital, The Chinese University of Hong Kong, Shatin, Hong Kong SAR China; 2 Li Ka Shing Institute of Health Sciences, The Prince of Wales Hospital, The Chinese University of Hong Kong, Hong Kong, Hong Kong SAR China; 3 University of Otago, 2 Riccarton Ave, Christchurch, 8140 New Zealand; 4 School of Physical and Chemical Sciences, University of Canterbury, Private Bag 4800, Christchurch, 8140 New Zealand; 5 The European Organization for Nuclear Research (CERN), Geneva, Switzerland; 6 MARS Bioimaging Ltd, 29a Clyde Rd, Christchurch, New Zealand; 7 Department of Radiology, Faculty of Medicine, The Prince of Wales Hospital, The Chinese University of Hong Kong, Shatin, Hong Kong SAR China

**Keywords:** Translational research, Imaging techniques, Osteoarthritis

## Abstract

To determine whether state-of-the-art multi-energy spectral photon-counting computed tomography (MARS) can detect knee arthroplasty implant failure not detected by standard pre-operative imaging techniques. A total knee arthroplasty (TKA) removed from a patient was reviewed. The extracted prosthesis [NexGen Legacy Posterior Stabilized (LPS) TKA] was analyzed as were pre-operative imaging examination and compared with a MARS-CT examination obtained of the extracted TKA prosthesis. Radiographs, fluoroscopy, ultrasound and MRI preoperatively did not reveal the cause of the implant failure. MARS CT images of the extracted prosthesis clearly showed the presence of posteromedial polyethylene and tibial tray wear which is compatible with the clinical appearance of the extracted TKA. MARS can identify polyethylene insert and metallic tibial tray wear as a cause of TKA failure, that could not be identified with on standard pre-operative imaging. Although clinical MARS CT system is still under development, this case does illustrate its potential clinical usefulness. This is the first study to document how MARS CT imaging can detect orthopedic implant failure not detected by standard current imaging techniques. This system has a potential clinical application in orthopedic patients.

## Introduction

Osteoarthritis is the single most common cause of disability in elderly adults and the knee is the most common involved site^1^. Symptomatic knee osteoarthritis is associated with pain, stiffness, and loss of mobility and can be effectively managed by total knee arthroplasty (TKA) when conservative management fails. In an aging society, TKA is in high demand with a projected 3.48 million TKA procedures performed annually in the United States alone by 2030^2^. Despite its frequent use, almost 20% of patients report suboptimal results after TKA with symptoms like persistent residual pain, stiffness and functional limitation^3^. Establishing the cause of symptoms and pain in patients with TKA is cardinal to proper management as revision surgery with an uncertain diagnosis has a poor outcome^4^.

Imaging is important in the diagnostic evaluation of TKA failure, mainly using modalities like radiography, fluoroscopy, ultrasound (US), computed tomography (CT), and magnetic resonance imaging (MRI). However, each of these modalities has its own limitations^5^. Radiography and fluoroscopy cannot delineate radiolucent structures like the surrounding soft-tissue structures or polyethylene-made insert or baseplate. US can assess effusions, and soft-tissue structures about the knee but cannot evaluate the prosthesis itself or the surrounding bony structure. CT is limited by beam-hardening artefact around the metallic femoral and tibial components, limiting visualization of the polyethylene insert as well as the immediate surrounding bone and soft tissue. Similarly, MRI is subject to metallic susceptibility obscuring the prosthesis and surrounding structures^5^.

The recently developed multi-energy spectral photon-counting computed tomography (MARS), by capturing the characteristic attenuation of different materials at multiple energy ranges^6,7^ with a conventional single X-ray source but equipped with multiple photon counting and energy resolving detectors, allows high efficiency detection within the human diagnostic energy range (30–120 keV). As each material has a measurable energy-dependent X-ray attenuation, spectroscopic imaging allows for multiple materials to be differentiated and quantified from each other simultaneously^8–11^. From this multi-energy data, a ‘color’ image of the object of interest can be generated, with high resolution structural information and also some compositional information^12–14^. Thereby material-specific images can be produced in MARS with minimal beam-hardening artefact often seen in standard CT imaging^15,16^.

The aim of this study was to demonstrate if MARS could detect knee arthroplasty implant failure that were not detected by current imaging techniques.

## Materials and methods

### Ethical statement

This study complied with the Declaration of Helsinki after obtaining approval from the Institutional Review Board of the local institution’s Research Ethical Committee [The Joint Chinese University of Hong Kong—New Territories East Cluster Clinical Research Ethics Committee (The Joint CUHK-NTEC CREC)] (CREC 2018.544). Experiments involving human participants (including the use of tissue samples) are performed with informed consent obtained.

### Prosthesis identification

A TKA prosthesis was identified from our extracted prosthesis bank with accompanying clinical records. The inclusion criteria for TKA prostheses were as follows: (1) TKA retrieved from symptomatic patient who had undergone revision surgery; (2) imaging and laboratory investigation had failed to identify the cause of TKA failure before revision surgery; and (3) the cause of failure could be ascertained based on morphologic assessment after extraction.

An extracted NexGen Legacy Posterior Stabilized (LPS) TKA (Zimmer Biomet, Warsaw, Indiana, USA) fulfilled these inclusion criteria and was used for analysis and imaging. This TKA consists of a femoral component, articular insert and tibial baseplate component. The femoral component was made of *Zimaloy* Cobalt-Chromium-Molybdenum Alloy. The articular insert was made of Ultra-High Molecular-Weight Polyethylene (UHMWPE) and the tibial baseplate was made of *Tivanium* Ti-6Al-4V Alloy. A small amount of cement and bone remained attached to the base of the femoral and tibial components after extraction.

### Clinical details and pre-revision imaging

The TKA belonged to an elderly female who had intermittent knee pain with clicking for 1 year. The TKA had been implanted twelve years earlier and she had remained well until her current knee symptoms started. There was no history of trauma, or symptoms of infection. Physical examination showed no signs of infection and full knee movement except for a distinct mid-range clunk. Serological inflammatory markers, white cell count and differential count were all normal.

Imaging of the TKA comprising radiographs (weight-bearing anteroposterior view, weight-bearing lateral view in extension, 30° flexion and 45° flexion, skyline view, lower-limb scanogram), fluoroscopy, ultrasound, and standard MRI on an 1.5 T imaging system was performed prior to revision surgery.

### Revision surgery

Revision TKA surgery was performed. During the operation, posteromedial polyethylene insert wear, posteromedial metallic tibial tray wear and severe metallosis were found. The whole prosthesis was removed, and extensive debridement was done. A constrained condylar knee arthroplasty was inserted. Intra-operative tissue culture was negative for infection.

### Imaging of extracted TKA prosthesis

The TKA protheses were imaged using a preclinical MARS scanner (MARS bioimaging Ltd., Christchurch, New Zealand)^7^. The scanner comprises a microfocus poly‐energetic X‐ray source and five Medipix 3RX energy‐resolving photon‐counting detectors within a continuous rotating gantry. A 2‐mm–thick cadmium zinc telluride sensor (14.1 × 14.1 mm^2^) bump bonded at 110 μm to a Medipix3RX readout chip was used^17,18^. MARS imaging of TKA specimens and calibration phantoms was performed using 5 charge‐summing mode energy thresholds of 7, 45, 55, 65, and 75 keV at 120 kVp (10). Imaging data were reconstructed simultaneously into non‐overlapping energy bins across the measured spectrum, such as 7–45.1, 45.1–54.9, 54.9–65.1, and 65.1–74.9 and 74.9 keV, at a spatial resolution of 0.09 × 0.09 × 0.09 mm. Images of the TKA prostheses were calibrated using mass attenuation coefficients estimated from the calibration phantom data to distinguish bone, cement, metallic femoral component and tibial baseplate and polyethylene insert and furnish material decomposition maps (see Supplementary File 1). The images obtained from MARS were then contrasted and compared with the results of previous imaging modalities obtained during clinical investigation.

## Results

### Pre-revision imaging of TKA prothesis

**Figure 1:**
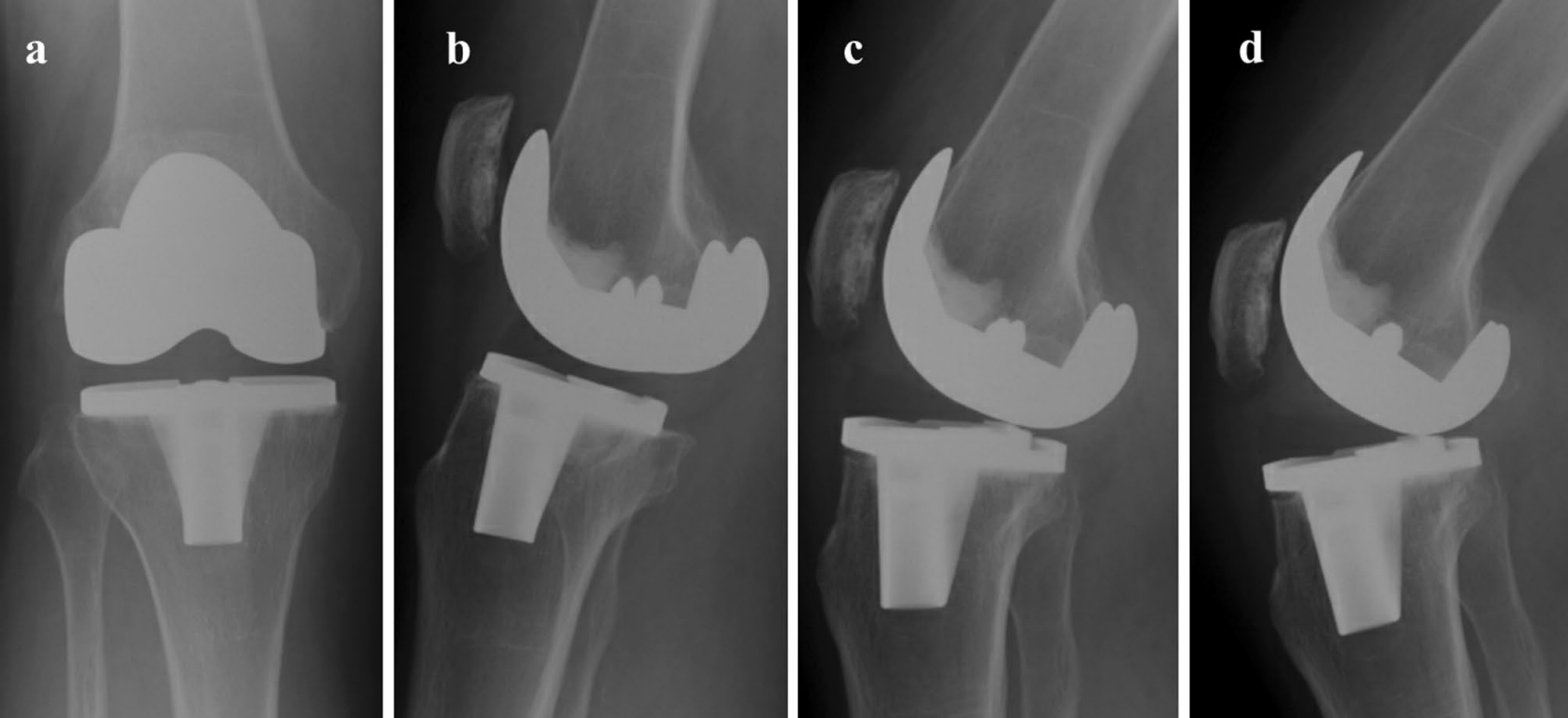
Weightbearing radiographs in (**a**) Anteroposterior extension (**b**) lateral extension, (**c**) lateral 30° flexion and (**d**) lateral 45° flexion weightbearing radiographs of knee. No definite polyethene thinning is evident.

**Figure 2:**
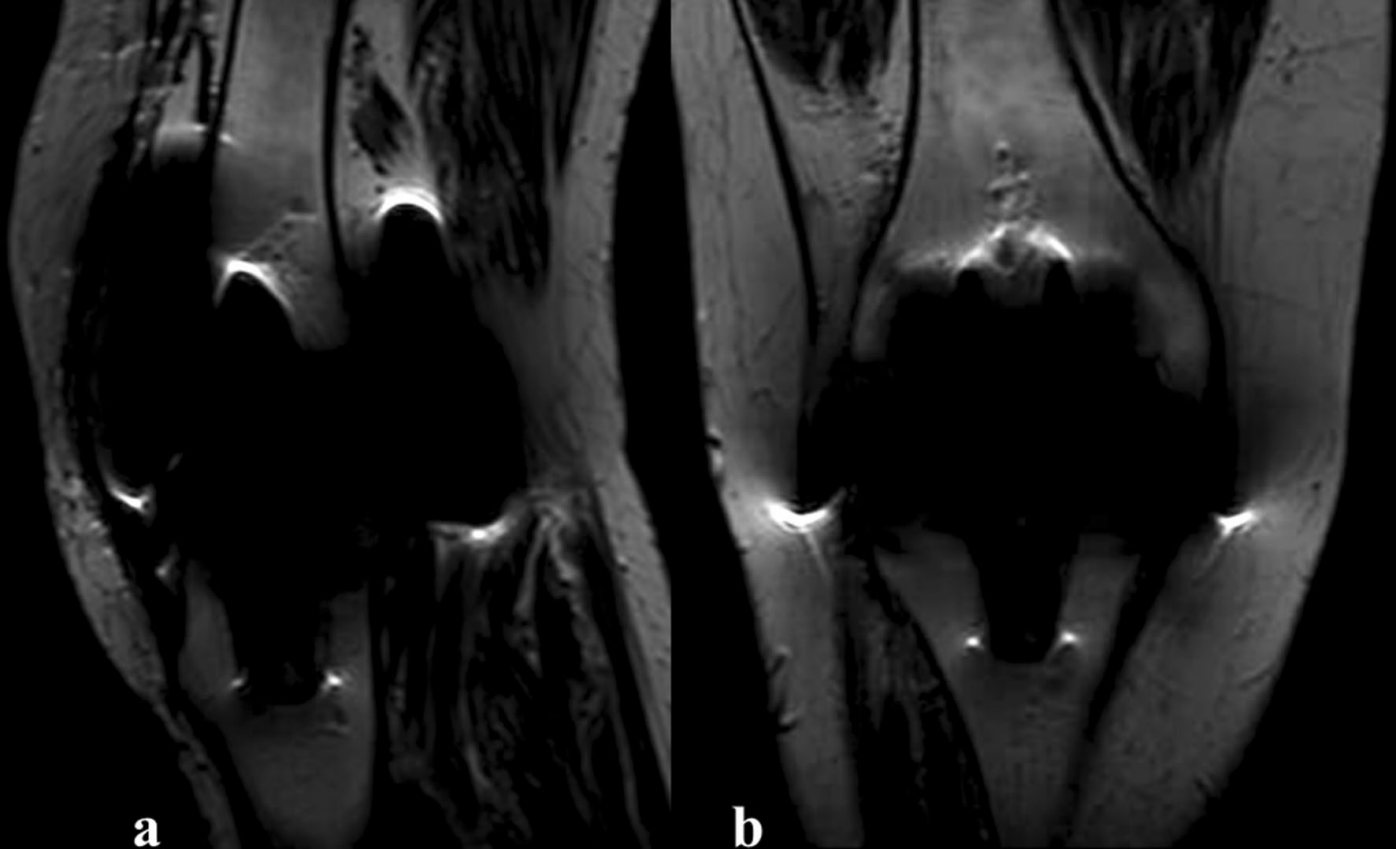
T1-weighted (**a**) sagittal and (**b**) coronal MRI images showing severe metallic artefact.

Radiographs of the knee were unremarkable (Fig. 1). Fluoroscopic screening noted a palpable clunk occurring during flexion–extension motion though but no discernible change in femorotibial relationship when this clunk occurred. No anteroposterior or varus-valgus instability was shown on fluoroscopy. Ultrasound revealed no evidence of iliotibial band syndrome or patellar clunk syndrome, with the clunk seeming to arise deeply from the tibiofemoral articulation rather than from the superficial structures. MRI revealed severe metallic artefact though was otherwise normal (Fig. 2).

### Post-revision imaging of TKA prosthesis with MARS

**Figure 3:**
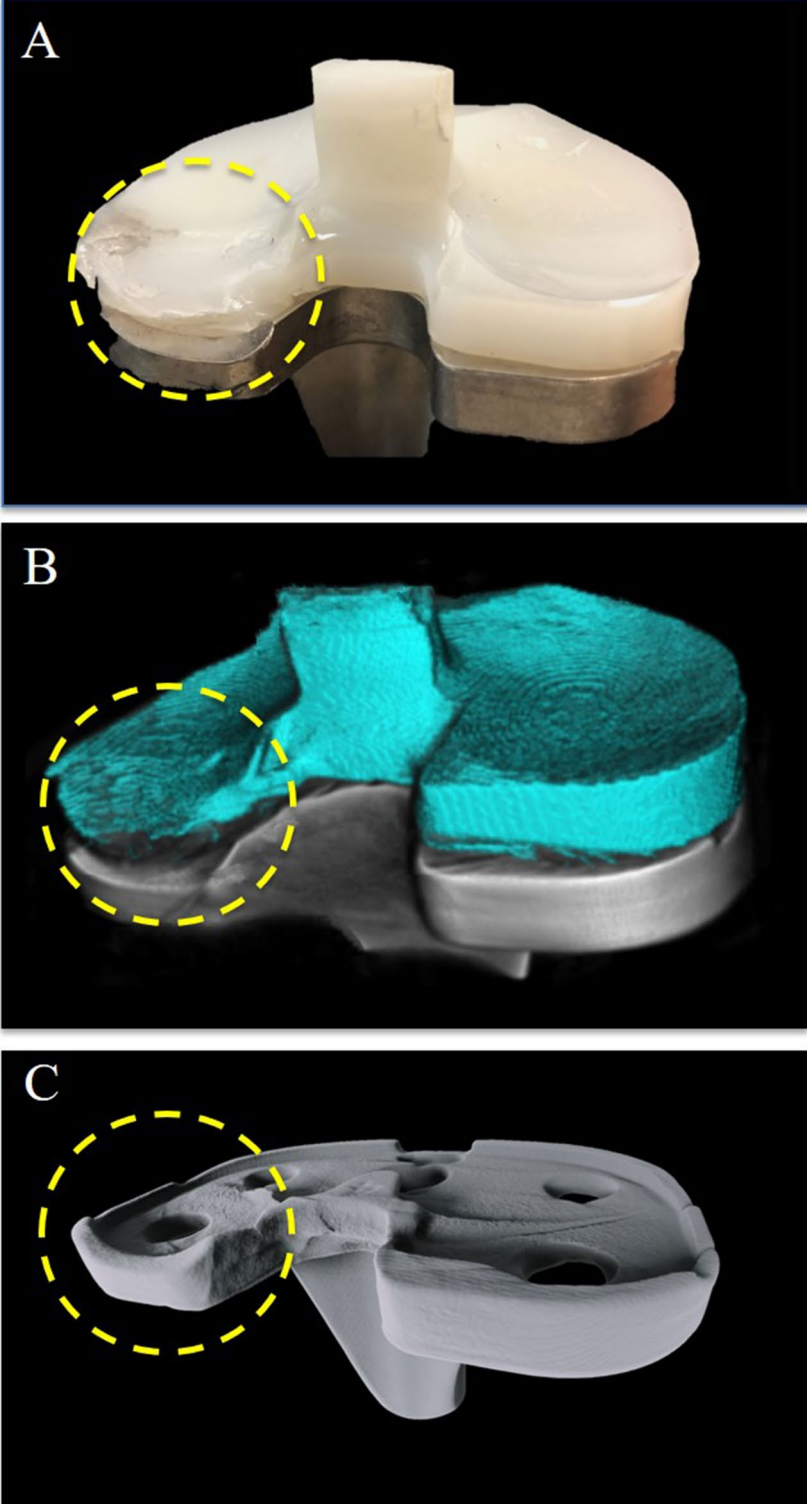
(**a**) Clinical photos, (**b**) MARS-CT images and (**c**) MARS-CT images with polyethelene digitally removed. The wear at the postomedial aspect of the polyethylene impant and tibial metallic tray is clearly visible (circle).

The MARS-acquired images clearly demonstrated clearly moderate-severity polyethylene insert wear, and posteromedial metallic tibial tray wear, compatible with the actual appearance of the prosthesis (Fig. 3) (Supplementary Video 1).

## Discussion

In the case presented, we showed how MARS-acquired CT images could clearly demonstrate polyethylene insert wear and metallic tibial tray wear clearly, not demonstrable by other imaging techniques highlighting the potential value of MARS imaging to aid clinical diagnosis.

Although TKA has long-term success in most cases, some cases do develop complications and fail over time. Common causes of TKA failure include aseptic loosening, instability, infection, polyethylene wear with or without osteolysis, metallosis and extensor mechanism failure^19^. If severe, these causes of failure are easy to identify on radiography by, for example, a fracture line in periprosthetic fracture, or periprosthetic radiolucency in aseptic loosening. However, in less severe cases, radiological changes are usually minimal or absent, as in the case presented where pre-operative imaging failed to identify the cause of pain and clicking. Multi-energy MARS CT was able to accurately reveal polyethylene wear without any significant metallic or other artefact characteristic of standard MRI^20^.

This has potential clinical implications. As the polyethylene insert wear was not identified early on and not exchanged, it continued to wear. Eventually, after the complete wear of part of the polyethylene insert, the underneath metallic tibial tray was exposed and underwent further wear due to abrasion by the metallic condyle of the femoral component. This produced metallosis that required extensive debridement. As the tibial tray and the femoral component were damaged, they had to be removed and replaced with a new implant which was of a constrained design to compensate the significant bone loss and instability created during the primary TKA removal. If the presented patient had been able to undergo MARS CT and identify polyethylene insert wear before its complete wear, the patient could have just undergone surgery that exchange the polyethylene insert, which is a much smaller scale surgery and preserve more bone and soft tissue of the patient.

This case illustrates the potential use of MARS to identify polyethylene wear early potentially enabling less extensive surgery to be performed, rather than major revision surgery with significant bone and soft tissue loss. This constitutes a significant difference to the patient care and medical cost. Based on our finding, MARS could play a unique role to gauge the remaining thickness of polyethylene insert before tibial tray is exposed. This would allow surgeons to determine the urgency of revision surgery and advise the patient the timing of revision.

There are limitations to this presentation. No arthrography or CT arthrography study was performed pre-operatively. These studies may have demonstrated the polyethylene wear^21^, though arthrography is, by definition, a minimally invasive procedure not to be undertaken lightly especially in the prosthetic knee where the risk of introducing infection is high. Although standard CT or MRI is currently not used to evaluate polyethylene wear due to artifact^22^, dual energy CT or MR imaging with metallic artifact reduction may have allowed a more in-depth assessment of the polyethylene implant though these techniques have not been evaluated in this regard. We supplement our study by imaging the extracted TKA prothesis with high-resolution peripheral quantitative computed tomography (HRpQCT) (XtremeCT II; Scanco Medical AG), which is another sophisticated form of CT and it could not demonstrate the polyethylene wear as shown by the MARS CT (see Supplementary File 2).

In conclusion, this is the first study to show how MARS CT imaging can detect orthopedic implant failure that is not detected by standard current imaging modalities. If confirmed in a clinical study, the accurate demonstration of polyethylene wear could have considerable implications of patient care, potentially allowing wear to be evaluated at an earlier stage, monitored accordingly with, if necessary, polyethylene insert replacement before metallic tray wear and metallosis occurs. Further cadaveric investigation is warranted to optimize imaging parameters and protocols, post-processing and analysis, which will lay the foundations for clinical study with clinical MARS.

## Supplementary Information

Supplementary Information 1.

Supplementary Information 2.

Supplementary Video 1.

## Data Availability

No datasets were generated or analysed during the current study.

## References

[1] Turkiewicz A (2014). Current and future impact of osteoarthritis on health care: A population-based study with projections to year 2032. Osteoarthr. Cartil..

[2] Kurtz S, Ong K, Lau E, Mowat F, Halpern M (2007). Projections of primary and revision hip and knee arthroplasty in the United States from 2005 to 2030. J. Bone Jt. Surg. Am..

[3] Bourne RB, Chesworth BM, Davis AM, Mahomed NN, Charron KD (2010). Patient satisfaction after total knee arthroplasty: Who is satisfied and who is not?. Clin. Orthop. Relat. Res..

[4] Mont MA, Serna FK, Krackow KA, Hungerford DS (1996). Exploration of radiographically normal total knee replacements for unexplained pain. Clin. Orthop. Relat. Res..

[5] Flierl MA, Sobh AH, Culp BM, Baker EA, Sporer SM (2019). Evaluation of the painful total knee arthroplasty. J. Am. Acad. Orthop. Surg..

[6] Taguchi K, Iwanczyk JS (2013). Vision 20/20: Single photon counting X-ray detectors in medical imaging. Med. Phys..

[7] Anderson NG, Butler AP (2014). Clinical applications of spectral molecular imaging: Potential and challenges. Contrast Media Mol. Imaging.

[8] Rajendran K (2017). Quantitative imaging of excised osteoarthritic cartilage using spectral CT. Eur. Radiol..

[9] Zainon R (2012). Spectral CT of carotid atherosclerotic plaque: Comparison with histology. Eur. Radiol..

[10] Ronaldson JP (2012). Toward quantifying the composition of soft tissues by spectral CT with Medipix3. Med. Phys..

[11] Stamp LK (2019). Clinical utility of multi-energy spectral photon-counting computed tomography in crystal arthritis. Arthritis Rheumatol..

[12] Butler, P. H. *et al.* In *Developments in X-Ray Tomography XII.* 111130C (International Society for Optics and Photonics).

[13] Bateman C (2018). MARS-MD: Rejection based image domain material decomposition. J. Instrum..

[14] Mandalika, V. B. H. A. C., Alexander, I., Billinghurst, M., Bartneck, C., Hurrell, M. A., De Ruiter, N., Butler, A. P. H., Butler, P. H. A hybrid 2D/3D user interface for radiological diagnosis. *J. Digit. Imaging* 56–73 (2018). 10.1007/s10278-017-0002-6 PMC5788824 28766028

[15] Amma, M. R. *et al.* In *Developments in X-Ray Tomography XII.* 111131D (International Society for Optics and Photonics).

[16] Rajendran K (2014). Reducing beam hardening effects and metal artefacts in spectral CT using Medipix3RX. J. Instrum..

[17] Ballabriga R (2013). The Medipix3RX: A high resolution, zero dead-time pixel detector readout chip allowing spectroscopic imaging. J. Instrum..

[18] Ballabriga R (2016). Review of hybrid pixel detector readout ASICs for spectroscopic X-ray imaging. J. Instrum..

[19] Mulcahy H, Chew FS (2014). Current concepts in knee replacement: Complications. AJR Am. J. Roentgenol..

[20] Hargreaves BA (2011). Metal-induced artifacts in MRI. AJR Am. J. Roentgenol..

[21] Hsu Y, Lin CH, Shu GHF, Hsieh TJ, Chen CK (2019). Fracture of the polyethylene tibial post in the posterior-stabilized total knee prosthesis: Arthrographic and CT arthrographic diagnosis. Skelet. Radiol..

[22] Expert Panel on Musculoskeletal, I. *et al.* ACR appropriateness criteria((R)) imaging after total knee arthroplasty. *J. Am. Coll. Radiol.* **14**, S421–S448, 10.1016/j.jacr.2017.08.036 (2017).

