# Supplementary material for: Multi-energy spectral photon-counting computed tomography (MARS) for detection of arthroplasty implant failure
Source: Sci Rep. 2021 Jan 15;11:1554. doi: 10.1038/s41598-020-80463-2 (PMC7810731; doi:10.1038/s41598-020-80463-2)
Supplement: Supplementary file 1 — Supplementary Information 1. [file 41598_2020_80463_MOESM1_ESM.pdf]

Supplementary file 1.

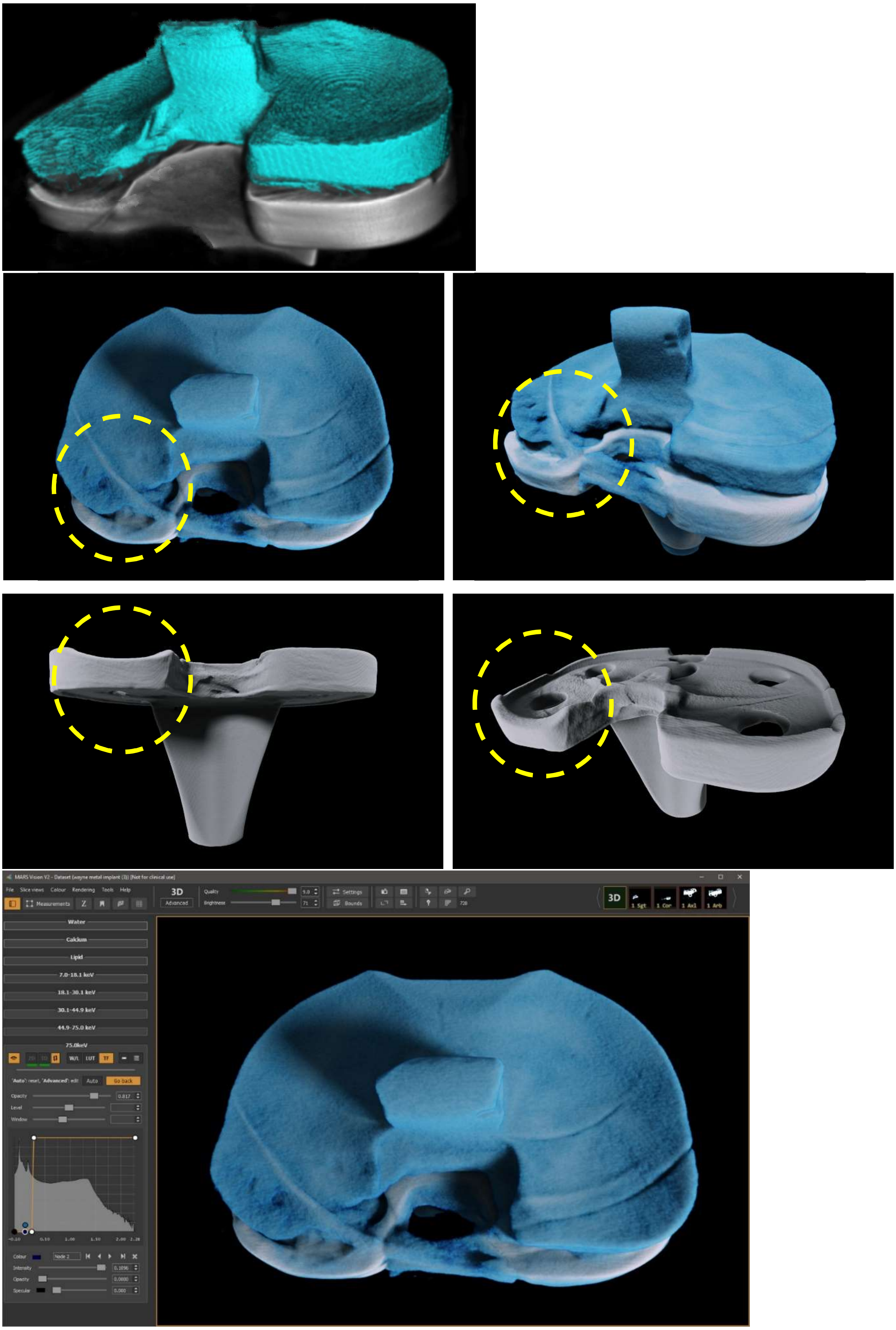

The images in Figure 3(b-c) were generated using MARS Vision by MARS Bioimaging Ltd [1,2,3] as shown in above picture. MARS Vision implements a modified version of a Monte-Carlo ray tracing direct volume renderer originally proposed by Kroes et al [4]. This volume rendering algorithm is computationally expensive, but it simulates realistic lighting and shadows to help improve depth perception. To generate the image in Figure 3 and the above pictures, we have manually identified the linear attenuation ranges that correspond to the various materials in the implant and assigned a unique colour/opacity gradient to each range by using a transfer function.

References:

[1] Mandalika et al (2017). A Hybrid 2D/3D User Interface for Radiological Diagnosis. Journal of Digital Imaging. 31. 10.1007/s10278-017-0002-6.

[2] Rajendran et al (2016). Quantitative imaging of excised osteoarthritic cartilage using spectral CT. European Radiology. 27. 10.1007/s00330-016-4374-7.

[3] Aamir et al (2014). MARS spectral molecular imaging of lamb tissue: data collection and image analysis. Journal of Instrumentation, Volume 9, February 2014.

[4] T. Kroes, F. Post & C. Botha. (2012). Exposure Render: An Interactive Photo-Realistic Volume Rendering Framework. PloS one. 7. e38586. 10.1371/journal.pone.0038586.
