# Supplementary material for: Multi-energy spectral photon-counting computed tomography (MARS) for detection of arthroplasty implant failure
Source: Sci Rep. 2021 Jan 15;11:1554. doi: 10.1038/s41598-020-80463-2 (PMC7810731; doi:10.1038/s41598-020-80463-2)
Supplement: Supplementary file 2 — Supplementary Information 2. [file 41598_2020_80463_MOESM2_ESM.pdf]

Supplementary file 2.

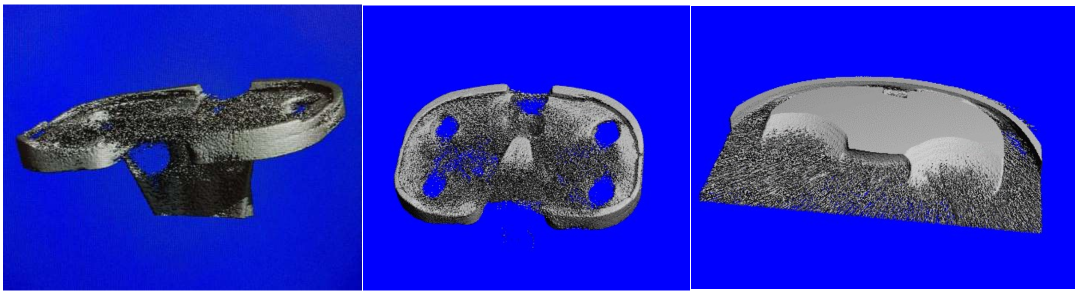

Above pictures showed the extracted TKA prothesis imaged by high-resolution peripheral quantitative computed tomography (HRpQCT) (XtremeCT II; Scanco Medical AG) and the polyethylene appeared transparent in the HRpQCT images. Therefore, HRpQCT cannot be used to identify polyethylene wear. We compared the performance of imaging with HRpQCT as it is another form of clinically used and advanced computed tomography technology. On the right side the table showed the imaging parameters set on the XtremeCT II.

**Parameters**	**XTremeCT II**
Energy (V)	59400
Current (µA)	900
FOV/Diameter (µm)	125952
Projections	750
Isotropic voxel size (µm)	82
Time (one stack) (seconds)	180
Total time (µs)	100000
Reconstruction-algorithm	Conebeam Conv./Backpr.
